# Anatomical and symptomatic outcomes in patients with Le Fort colpocleisis with or without hysterectomy

**DOI:** 10.1186/s12905-022-01868-x

**Published:** 2022-07-09

**Authors:** Mehmet Ferdi Kinci, Burak Sezgin, Mehmet Onur Arslaner, Deniz Akin Gökbel, İsmail Gökbel, Ahmet Akın Sivaslioğlu

**Affiliations:** 1 Obstetrics and Gynecology Department, Muğla University Education and Research Hospital, Muğla, Turkey; 2 Obstetrics and Gynecology Department, Menteşe State Hospital, Muğla, Turkey

**Keywords:** De Novo urinary incontinence, Le Forte colpocleisis, Lower urinary tract symptoms, Pelvic organ prolapse

## Abstract

**Background:**

We aimed to evaluate the short-term anatomical and clinical outcomes of elderly patients who underwent the Le Fort colpocleisis operation due to pelvic organ prolapse (POP) in our clinic.

**Methods:**

The medical records of fifty-nine sexually inactive females, with stage 2 or higher vaginal or uterine prolapse who underwent Le Fort colpocleisis operations were prospectively analysed. Preoperative and 12th month postoperative data were recorded. Lower urinary tract symptoms (LUTS) was also evaluated preoperatively and 12 months postoperatively in all patients. Anatomical success was determined as no prolapse of any POP-Q point at or below 1 cm above vaginal introitus.

**Results:**

A total of 59 patients were included in this study. The average age of the patients was 71.67 ± 7.01 (years). The mean BMI was 27.1 ± 9.52 kg/m^2^. POP-Q point, C (6.70 ± 2.44 vs. − 2.66 ± 1.21) measurements were significantly deeper, as well as Gh (4.83 ± 0.94 vs. 4.26 ± 0.94) and TVL (3.51 ± 1.24 vs. 8.93 ± 1.73) measurements were significantly higher after surgery than during the preoperative period (*p* < 0.01, *p* < 0.01, *p* < 0.01, respectively). There were no cases of recurrence. The evaluation of LUTS at the 12-months postoperative follow-up revealed significant differences for SUI, urinary frequency, nocturia, and pelvic pain symptoms (*p* = 0.007, *p* < 0.001, *p* = 0.01, *p* < 0.001, respectively).

**Conclusions:**

Le Fort colpocleisis is a simple and effective procedure that provides successful anatomical and clinical outcomes in sexually inactive and elderly women with POP. However, the long-term results of this procedure need further investigation.

## Background

The incidence of surgically managed pelvic organ prolapse (POP) ranges from 1.5 to 49/10000, although several factors may influence its incidence, especially age [1]. According to several studies, women have an estimated 10–20% risk of undergoing POP repair in their lifetime [2, 3]. As a result of the increasing life expectancy, a steadily increasing number of POP surgeries are likely to requested by gynaecological patients.

POP surgeries are mainly classified into reconstructive and obliterative methods [2]. The decision on the surgical method depends on a plethora of factors, including patient age, overall condition, comorbid disease status, POP severity, and sexual life. Le Fort colpocleisis is one of the obliterative modalities preferred in elderly patients who are no longer sexually active.

Le Fort colpocleisis was first described in 1877 by Leon Le Fort as a surgical procedure that basically involves closing the vagina wherein the pubocervical fascia (PCF) and the rectovaginal fascia (RVF) are approximated and sown along the midline. Anatomical success rates up to 98% have been reported [4].

In the literature, there are some studies about Le fort colpocleisis that simultaneously present anatomical and clinical results. In this study, we prospectively analysed Le Fort colpocleisis operations performed in our department. Here, we evaluated the anatomical outcomes of the surgery using the pelvic organ prolapse quantification (POP-Q) tool in addition to the clinical outcomes, which were assessed by the lower urinary tract symptoms (LUTS).

## Methods

### Study design

The study included 59 patients who were diagnosed with vaginal or utero-vaginal prolapse and therefore underwent Le Forte colpocleisis either alone or in combination with other gynaecological surgeries between January 2016 and October 2020. This study was approved by the Mugla University Clinical Research Ethics Committee (Ethics approval date: Jul 02, 2020, No: 07/VI). As a part of the preoperative procedure, a detailed medical history was retrieved, and gynaecological examination was performed in all patients. Before the operation, all the patients were counselled about the desire for future vaginal intercourse. According to the counselling results, the surgery was planned for patients with no intention to have any sexual intercourse. Preoperative and postoperative outpatient follow-up examinations as well as the surgeries were all performed by two surgeons experienced in urogynecology (AAS and MFK). We didn’t perform any urodynamic tests because of our clinics daily practice. Informed consent was obtained from all participants.

When patients were scheduled for surgery, queries were conducted to record age, gravidity, parity, body mass index (BMI), comorbid diseases, history of previous surgeries, and LUTS questionnaire. LUTS questionnaire that is part of our routine urogynecological evaluations in our clinic, such as stress urinary incontinence (SUI), frequency, urgency, intermittency, nocturia, pelvic pain, emptying. Prolapse was graded using the POP-Q classification during gynaecological examination [5]. Complications were evaluated according to the Clavien–Dindo classification and classified using the joint project of the International Continence Society and the International Urogynecological Association Prosthesis/Graft Complication Classification System [6, 7].

### The surgery

All patients were placed in the lithotomy position. Then, spinal anaesthesia was performed. A rectangular incision was made on the anterior aspect of the vaginal wall starting at 0.5 cm distal to the urethral orifice and extending until 1 cm was left before reaching the anterior lip of the uterine cervix. The vaginal mucosa was stripped from the PCF. Then, the posterior vaginal wall was also excised in a rectangular shape, starting from the insertion point of the uterosacral ligaments and extending until 1 cm was left before the posterior cervical lip. Vaginal mucosa was denuded from the RVF. The edges of the lateral residual vaginal mucosal walls were tied using no: 2/0 polyglactin suture forming a drain tunnel. Drain tunnels were performed for all patient. Afterwards, the PCF at the anterior wall and RVF at the posterior wall were aligned along the vertical plane. Then, 2/0 polyglactin was used to suture the PCF to the RVF for vaginal closure.

### Postoperative follow-up

The urinary catheter was retracted after the mobilization, and a complete blood count sample was collected 24 h after the surgery. Patients were discharged to home at 48 h, provided no complications had occurred. All the patients were examinated at postoperative 10th day after the surgery for short term complications. No complication was observed. All patients were examined at postoperative months 1 and 12. During the follow-up conducted at our outpatient department 12 months after the surgery, LUTS that was administered preoperatively was readministered, and the anatomical marker of total vaginal length (TVL) and genital hiatus (Gh) as well as the location called the C point, which were the measurement parameters included in the POP-Q scoring system, were re-evaluated (Table 1). TVL and C point measured from the drain tunnel with elastic ruler.

**Table 1:** Comparison of pre-operative and post-operative POP-Q findings

	Pre-operative	Post-operative month 12	Paired differences (95%CI)	*p*
Aa	2.23 ± 1.00	NA	NA	< 0.001*
Ap	2.40 ± 0.81	NA	NA	
Ba	6.50 ± 2.02	NA	NA	
Bp	6.30 ± 1.96	NA	NA	
C	6.70 ± 2.44	− 2.66 ± 1.21	9.36 ± 2.42 (8.46–10.27)	
TVL	8.93 ± 1.73	3.51 ± 1.24	5.41 ± 2.32 (4.53–6.29)	
Gh	4.83 ± 0.94	4.26 ± 0.94	0.56 ± 0.72 (0.29–0.83)	

*NA* non-applicable

*p < 0.05, as statistically significant

### Statistical analysis

Statistical analysis was carried out using SPSS software (IBM SPSS Statistics, Version 22.0. Armonk, NY: IBM Corp). The Shapiro–Wilk test was applied to assess the distribution of variances. Variables with a normal distribution are presented as the mean ± standard deviation (SD). The comparison of preoperative and postoperative haemoglobin (Hb) values was completed using a paired-samples t test. The relationship of the LUTS between the preoperative and postoperative groups was analysed using the chi-squared test. Values of *p* < 0.05 were accepted as statistically significant.

## Results

A total of 59 patients were included in the study. The average age of the patients was 71.67 ± 7.01 (years). These women had a parity of 3.20 ± 1.12, on average. The mean BMI was 27.1 ± 9.52 kg/m^2^. Thirty-four patients had a prior surgical history that included a vaginal hysterectomy, vaginal hysterectomy + culdoplasty in three patients. Vaginal hysterectomy in combination with colpocleisis was performed in 22 patients, while 37 patients with a surgical history of previous hysterectomy underwent colpocleisis alone.

The patients’ preoperative mean Hb value was 12.95 ± 1.54 g/dL compared to a postoperative value of 11.01 ± 1.25 g/dL. The mean Hb change was 1.94 ± 0.29 g/dL.

When manifestations of preoperative versus postoperative anatomical status were evaluated, C (6.70 ± 2.44 vs. − 2.66 ± 1.21) measurements were significantly deeper, as well as Gh (4.83 ± 0.94 vs. 4.26 ± 0.94) and TVL (3.51 ± 1.24 vs. 8.93 ± 1.73) measurements were significantly higher after surgery than during the postoperative period (*p* < 0.01, *p* < 0.01, *p* < 0.01, respectively) (Table 1).

When manifestations of preoperative versus postoperative symptoms were evaluated, significant differences were found for SUI, frequency, nocturia and pelvic pain during the postoperative period (*p* = 0.007, *p* < 0.001, *p* = 0.01, *p* < 0.001, respectively) (Table 2).

**Table 2:** Pre-operative and post-operative (month 12) lower urinary tract symptoms

Lower urinary tract symptoms	Pre-operative			Post-operative month 12			*p* Value
		N (59)	%		N (59)	%	
Intermittency	No	47	79.66	No	58	98.3	0.11
	Yes	12	20.33	Yes	1	1.69	
Frequency	No	31	52.54	No	57	96.61	< 0.001*
	Yes	28	47.45	Yes	2	3.38	
Urgency	No	47	79.66	No	58	98.3	0.11
	Yes	12	20.33	Yes	1	1.69	
Nocturia	No	40	67.79	No	56	94.91	0.01*
	Yes	19	32.2	Yes	3	5.08	
Pelvic pain	No	31	52.54	No	57	96.61	< 0.001*
	Yes	28	47.45	Yes	2	3.38	
Difficulty emptying	No	47	79.66	No	56	94.91	0.254
	Yes	12	20.33	Yes	3	5.08	
Stress urinary incontinence	No	29	49.15	No	47	79.66	0.007*
	Yes	30	50.84	Yes	12	20.33	

*p < 0.05, as statistically significant

When postoperative complications were classified by severity according to the Clavien–Dindo classification, grade 1 complications [5.08%—urinary tract infection (UTI)] were noted in three patients, and grade 2 complications were noted (3.38%—atelectasis) in two patients.

## Discussion

Le Forte colpocleisis is not only an effective surgical treatment for patients with POP but is also favourable for a short operative time and low risk of associated complications and morbidity [8, 9]. Colpocleisis is preferred in elderly and fragile patients who are no longer sexually active. In this study, we compared the POP-Q and LUTS as detected prior to surgery and 12 months after surgery in patients who underwent Le Forte colpocleisis in our department. Our analysis revealed statistically significant changes in point C, TVL, and Gh measurements at postoperative month 12 compared to the preoperative measurements as a result of surgery. Although these changes seem clinically insignificant, this result may indicate anatomical recovery. Moreover, among the LUTS, we found statistically significant improvement in SUI, frequency, nocturia which implies a positive impact on functional recovery.

Le Forte colpocleisis is mostly applied in elderly women. To this end, the mean age of 325 patients included in the study of Zebede et al. was 81.36 ± 5.3 years, and likewise, in the study by Reisenauer et al., 58 patients had a mean age of 81.9 ± 6.4 years [4, 10]. In our cohort, the mean age was 71.67 ± 7.01 years.

According to the literature, Le Forte colpocleisis features as a low-risk and well-tolerated surgery [11]. The reasons for these features are most likely due to the easy surgical technique and the short operative time than vaginal hysterectomy, sacrocolpopexy and other obliterative methods [12–14]. Many publications have reported intraoperative and postoperative complications. According to the review by FitzGerald et al., postoperative cardiac, thromboembolic, pulmonary, and cerebrovascular complications were detected in approximately 5% of patients. On the other hand, approximately 15% of the patients experience minor complications, including UTI, vaginal haematoma, cystotomy, fever, and thrombophlebitis [15]. Another study conducted by Hullfish et al. [16] detected peri-operative complications in 18 out of 94 patients, four patients experienced the most frequent complication which was UTI. In our study, a major complication occurred in only one patient (1.69%) who developed atelectasis, and a minor complication occurred in two patients (3.38%), both of whom had UTI. The lower rates of major and minor complications experienced in our study are most likely associated with the younger patients in our sample.

The POP-Q scoring system is one of the most widely used methods worldwide for the classification of POP and its relevant surgical success [17]. Following a successful colpocleisis, the POP-Q scores should improve remarkably. In their study by Reisenauer et al. [10], POP-Q scores were evaluated in 37 patients. In the same study, the mean measurement for TVL was 22 ± 9 mm, for PB was 45 ± 13 mm, and for Gh was 17 ± 6 mm. In another study conducted by Fitzgerald et al. [15], TVL, Gh, and PB were evaluated preoperatively and postoperatively. In chronological order of their measurements, the mean values for TVL were 9 cm and 3 cm; for Gh were 6 cm and 2 cm; and for PB were 3 cm and 4 cm. In our study, the preoperative vs. postoperative mean values for TVL were 8.93 ± 1.73 mm vs*.* 35.1 ± 12.4 mm, for Gh were 4.83 ± 0.94 mm versus 4.26 ± 0.94 mm, and for C point were 6.70 ± 2.44 mm versus − 2.66 ± 1.21 mm, respectively. We believe that the anatomical improvement in TVL develops from the sutures placed on the vertical axis and that the improvement in the C point is a result of vertical closure performed along the free edges of the vaginal apex.

Along with anatomical improvement, functional improvement is also essential following POP surgery. Various questionnaires are often used to investigate this aspect, such as those on LUTS. In a study by Neimark et al. [18], 45 women underwent Le forte colpocleisis, high perineoplasty and received tension-free vaginal tape (TVT). When the preoperative and postoperative 3^th^ month results of these cases were evaluated, SUI and postvoiding residual symptoms were significantly decreased, and constipation and irritative voiding symptoms did not change. In addition, the patients were administered the postoperative QoL questionnaires, but it was not possible to make a healthy evaluation because the questionnaire was not administered preoperatively. In the study of Hullfish et al. [16], patient queries were performed after an average of 2.75 years following the surgery. Accordingly, 34 women who had preoperative urgency and frequent symptoms were asked whether they considered their condition to have improved, two (5.9%) were not sure, one (2.9%) disagreed, and the remaining women agreed. Furthermore, of the 34 women who had preoperative difficulty emptying their bladder, four (11.8%) were not sure, and another four (11.8%) disagreed. In contrast to Hullfish et al.’s [16] study, our study did not provide a rank of options, but instead yes/no options for SUI, frequency, difficulty of urination, and bulging of the vagina improved by 12 months postoperatively, as stated by our respondents. In addition to the difference in questionnaire methodology, compared to their results, our findings also indicate a higher rate of patient satisfaction. Koski et al. [19] conducted the Urinary Distress Inventory-6 on 21 women 9.3 months after Le Fort colpocleisis on average. According to the answers provided by the respondents, 7 (%33.3) women had frequent urination and urge incontinence, 5 (23.8) women had SUI, and 4 (19%) women had small amount of leakage and difficulty emptying. In the results of our study, 2 (3.38%) woman had frequency and pelvic pain, 1 (%1.69) had urge incontinence and intermittency, 12 (20.33%) women had SUI, and 3 (5.08%) women had difficulty emptying and nocturia. The reason underlying the improvement in LUTS may be the anatomical elimination of anterior or posterior vaginal compartment defects.

SUI affects the social life and physical activities of women negatively, particularly those of advanced age. Its incidence varies due to a number of factors; the incidence ranges from 16.1 to 68.8% [20]. The study by Fitzgerald et al. [21] included 152 patients who underwent colpocleisis, of whom 54% had preoperative SUI that was reduced to 15% postoperatively. Glavind et al. [22] investigated 40 patients for SUI, of whom 17 (42.5%) had SUI during the preoperative period, while 7 (17.5%) patients persisted and one (2.5%) patient had increased SUI during the postoperative period. In our patients, preoperative SUI was detected in 50.84%, while 20.33% had SUI at postoperative month 12. The results of the current study are comparable to the results documented in the literature. Augmented support to the urethral neck is likely the reason for the improvement we detected in SUI. On the other hand, de novo SUI developed in only one patient.

The entire sample of patients underwent surgery in the same centre by two surgeons experienced in their field who also followed up the patients postoperatively. This helped to standardized the study data. A strong aspect of this study is that it addresses not only anatomic improvement but also improvement in LUTS.

## Limitations

The study is limited by its single-centre design and small sample size. The other limitations are the lack of comparison with other procedures and absence of quality of life questionnaires.

## Conclusion

Le Fort colpocleisis is known as a surgery with a high rate of anatomical and functional successes in the short term and a low risk of complications especially in elderly patients with POP who are no longer sexually active. In addition to anatomical improvement, the improvement we have achieved in LUTS implies that it also provides clinical recovery. To further demonstrate such recovery, studies with a larger sample size from different backgrounds and long follow-up periods are needed.

## Data Availability

The datasets used and/or analysed during the current study available from the corresponding author on reasonable request.

## Abbreviations

POP: Pelvic organ prolapse
GPFBQ: Global Pelvic Floor Bother Questionnaire
PCF: Pubocervical fascia
RVF: Rectovaginal facia
POP-Q: Pelvic organ prolapse quantification
BMI: Body mass index
LUTS: Lower urinary tract symptoms
SUI: Stress urinary incontinence
TVL: Total vaginal length
Gh: Genital hiatus
Hb: Hemoglobin
UTI: Urinary tract infection
TVT: Tension-free vaginal tape
